# Supplementary material for: Probiotic Bifidobacterium breve MCC1274 Mitigates Alzheimer’s Disease-Related Pathologies in Wild-Type Mice
Source: Nutrients. 2022 Jun 19;14(12):2543. doi: 10.3390/nu14122543 (PMC9231139; doi:10.3390/nu14122543)
Supplement: Supplementary file 1 [file nutrients-14-02543-s001.zip › nutrients-1762282-supplementary.pdf]

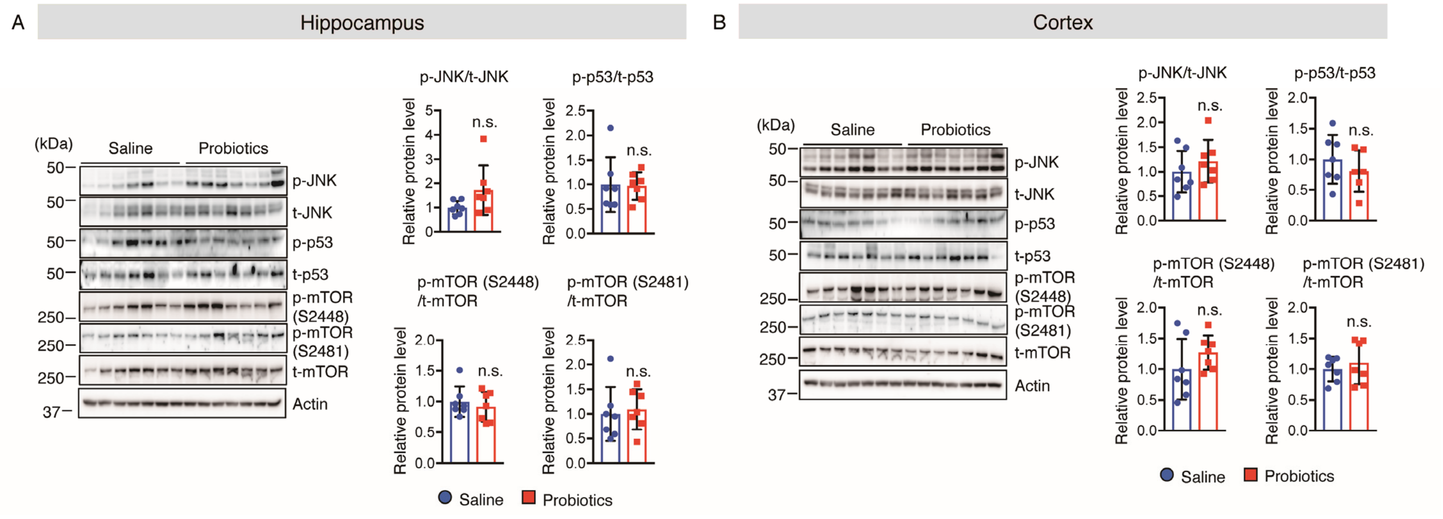

Supplementary Figure S1: Oral supplementation of *B. breve* MCC1274 does not affect JNK, P53, and mTOR protein levels in both the hippocampus and cortex of WT mice. Western blot analysis of phosphorylated (p-) JNK, total (t-) JNK, p-p53, t-p53, p-mTOR, t-mTOR, and actin in the hippocampus (A) and cortex (B). The protein levels were quantified by densitometry, normalized to the actin level, and expressed as a relative protein level. Data are represented as mean ± SD, n = 7 per group, n.s. no significant difference compared with the saline group, as determined by Student's *t*-test.

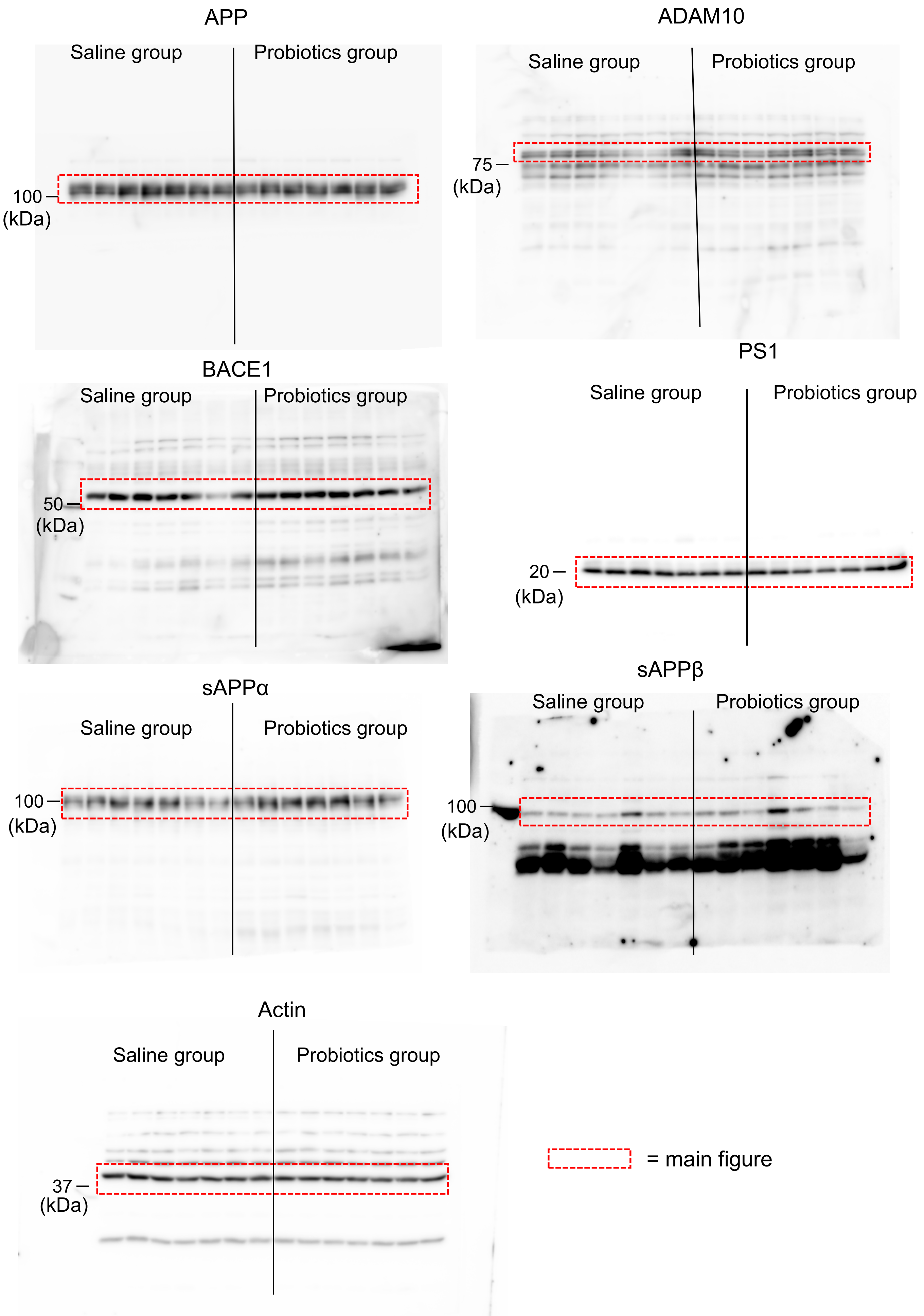
APP
Saline group
Probiotics group
100
(kDa)
ADAM10
Saline group
Probiotics group
75
(kDa)
BACE1
Saline group
Probiotics group
50
(kDa)
PS1
Saline group
Probiotics group
20
(kDa)
sAPPα
Saline group
Probiotics group
100
(kDa)
sAPPβ
Saline group
Probiotics group
100
(kDa)
Actin
Saline group
Probiotics group
37
(kDa)
= main figure

Figure 3 A

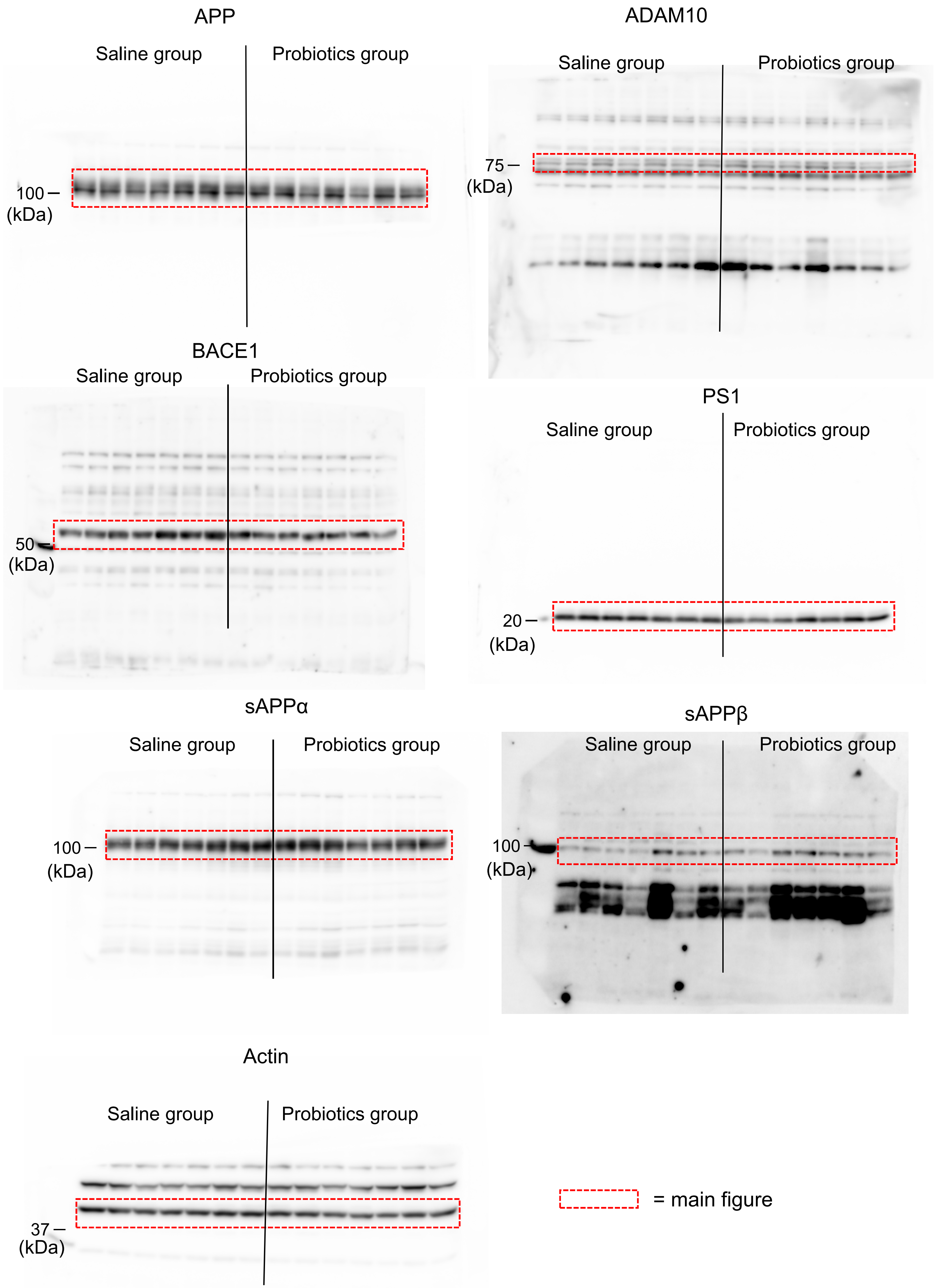
APP
Saline group
Probiotics group
100
(kDa)
ADAM10
Saline group
Probiotics group
75
(kDa)
BACE1
Saline group
Probiotics group
50
(kDa)
PS1
Saline group
Probiotics group
20
(kDa)
sAPPα
Saline group
Probiotics group
100
(kDa)
sAPPβ
Saline group
Probiotics group
100
(kDa)
Actin
Saline group
Probiotics group
37
(kDa)
= main figure

Figure 3 B

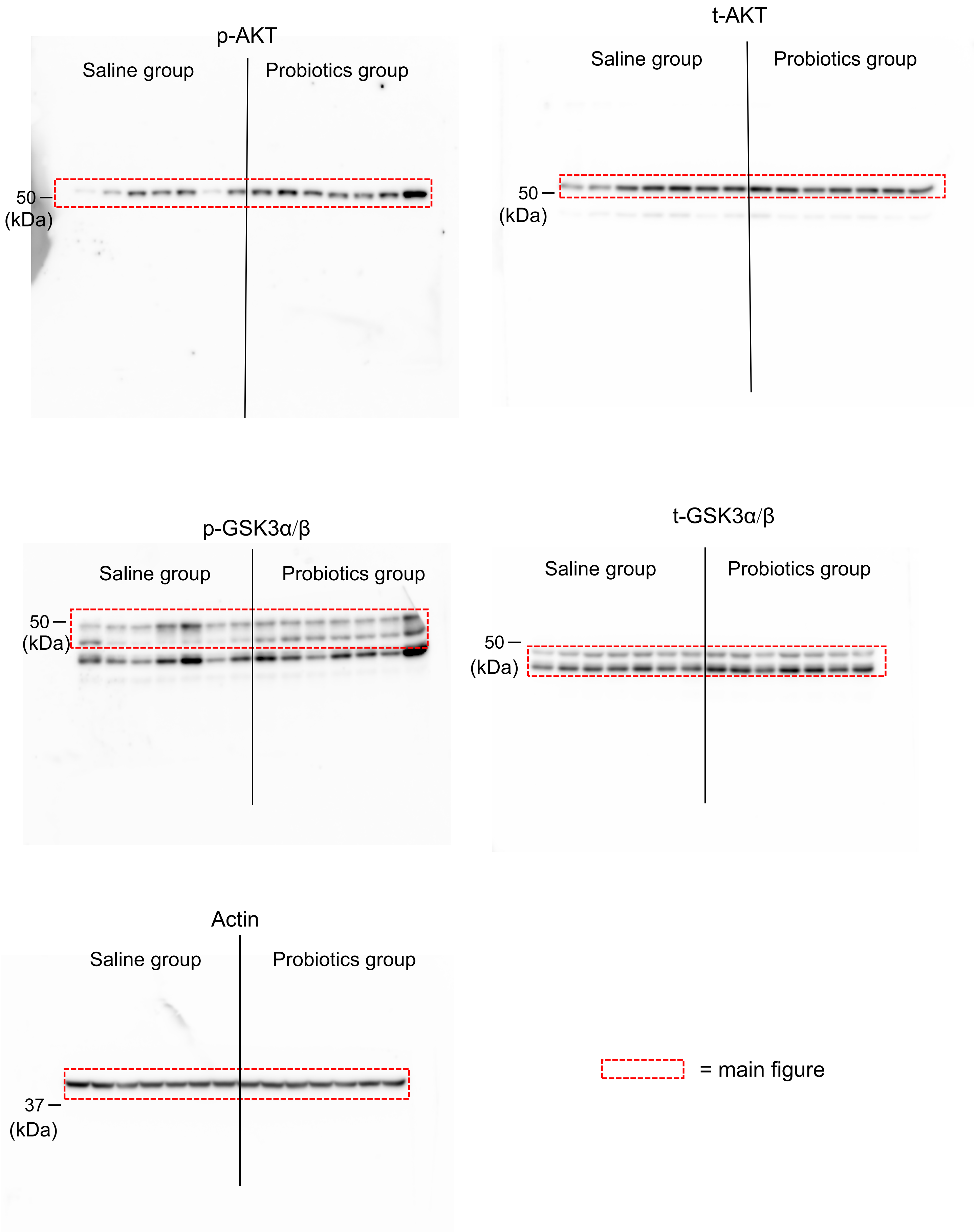
p-AKT
Saline group
Probiotics group
50
(kDa)
t-AKT
Saline group
Probiotics group
50
(kDa)
p-GSK3α/β
Saline group
Probiotics group
50
(kDa)
t-GSK3α/β
Saline group
Probiotics group
50
(kDa)
Actin
Saline group
Probiotics group
37
(kDa)
= main figure

Figure 4 A

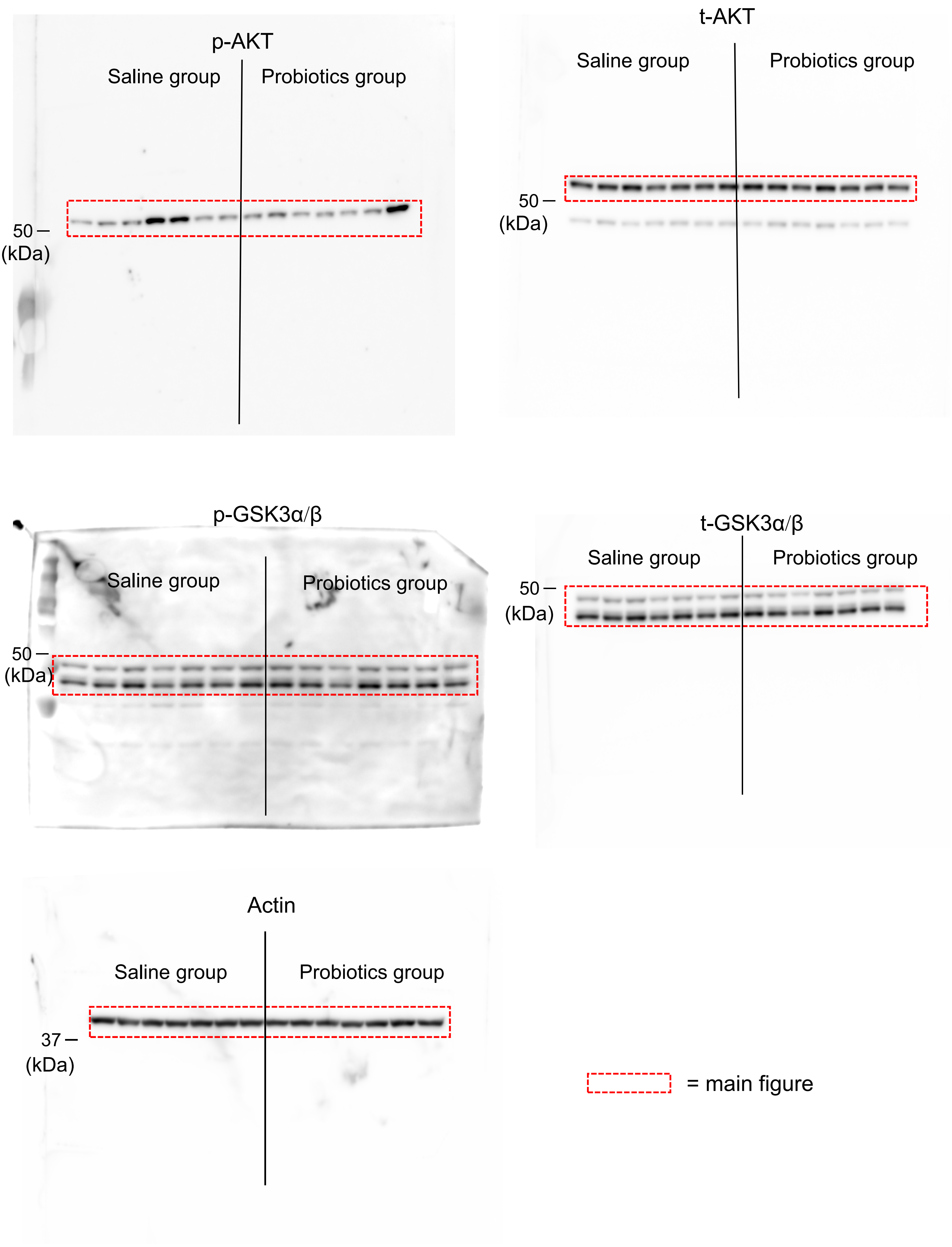
p-AKT
Saline group
Probiotics group
50
(kDa)
t-AKT
Saline group
Probiotics group
50
(kDa)
p-GSK3α/β
Saline group
Probiotics group
50
(kDa)
t-GSK3α/β
Saline group
Probiotics group
50
(kDa)
Actin
Saline group
Probiotics group
37
(kDa)
= main figure

Figure 4 B

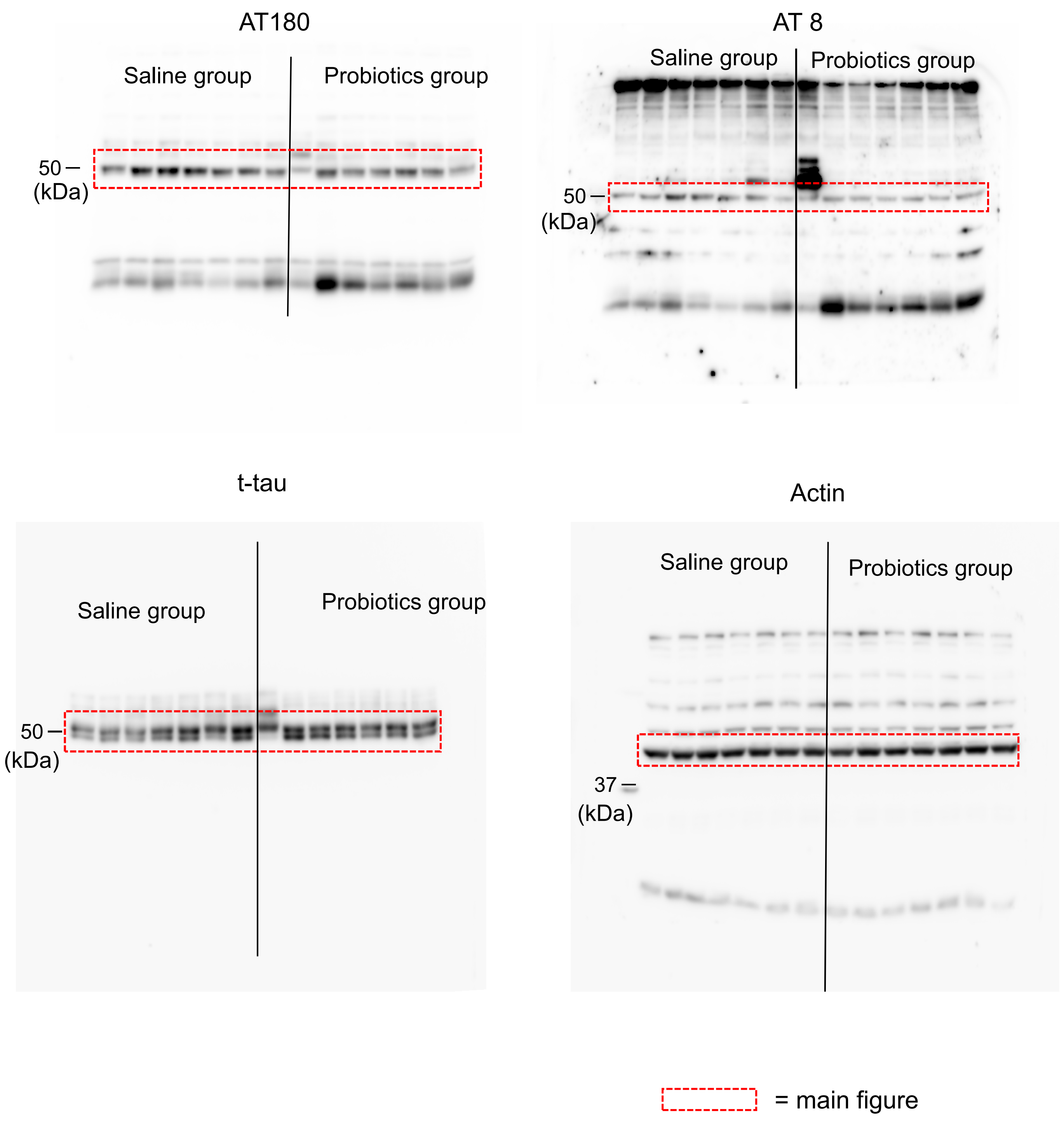
AT180
Saline group
Probiotics group
50
(kDa)
AT 8
Saline group
Probiotics group
50
(kDa)
t-tau
Saline group
Probiotics group
50
(kDa)
Actin
Saline group
Probiotics group
37
(kDa)
= main figure

Figure 5 A

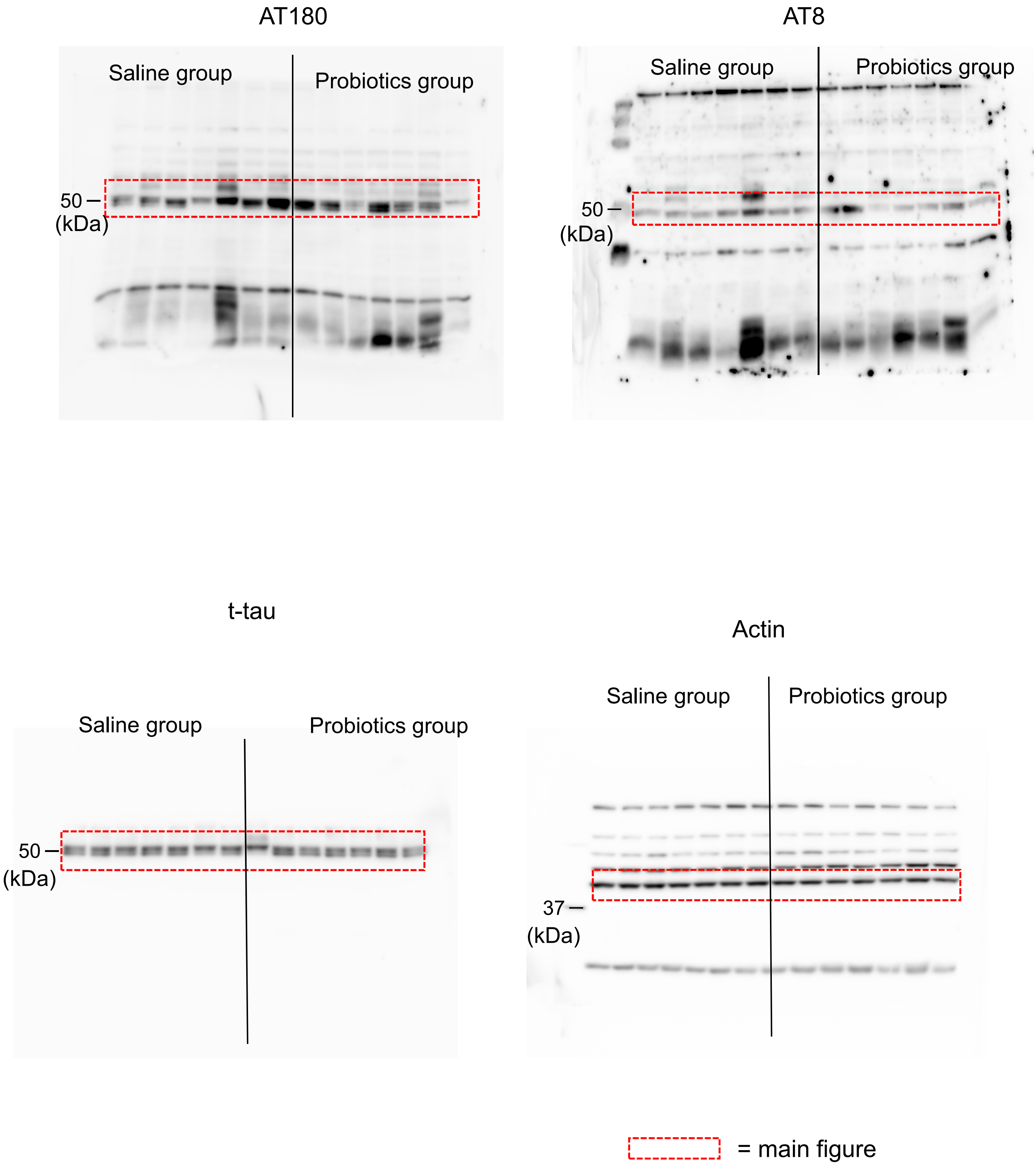
AT180
Saline group
Probiotics group
50
(kDa)
AT8
Saline group
Probiotics group
50
(kDa)
t-tau
Saline group
Probiotics group
50
(kDa)
Actin
Saline group
Probiotics group
37
(kDa)
= main figure

Figure 5 B

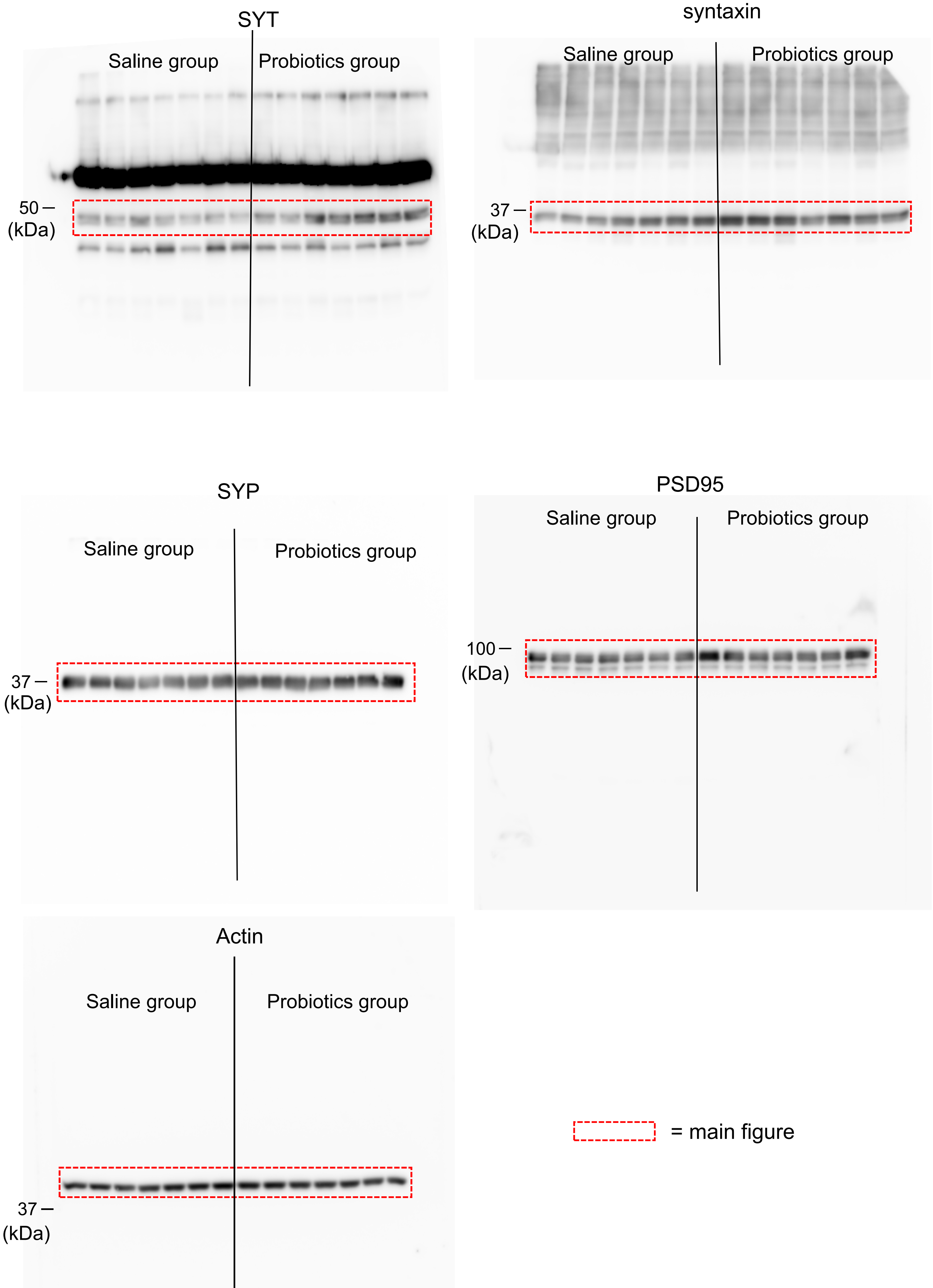
SYT
Saline group
Probiotics group
50 —
(kDa)
syntaxin
Saline group
Probiotics group
37 —
(kDa)
SYP
Saline group
Probiotics group
37 —
(kDa)
PSD95
Saline group
Probiotics group
100 —
(kDa)
Actin
Saline group
Probiotics group
37 —
(kDa)
= main figure

Figure 6 A

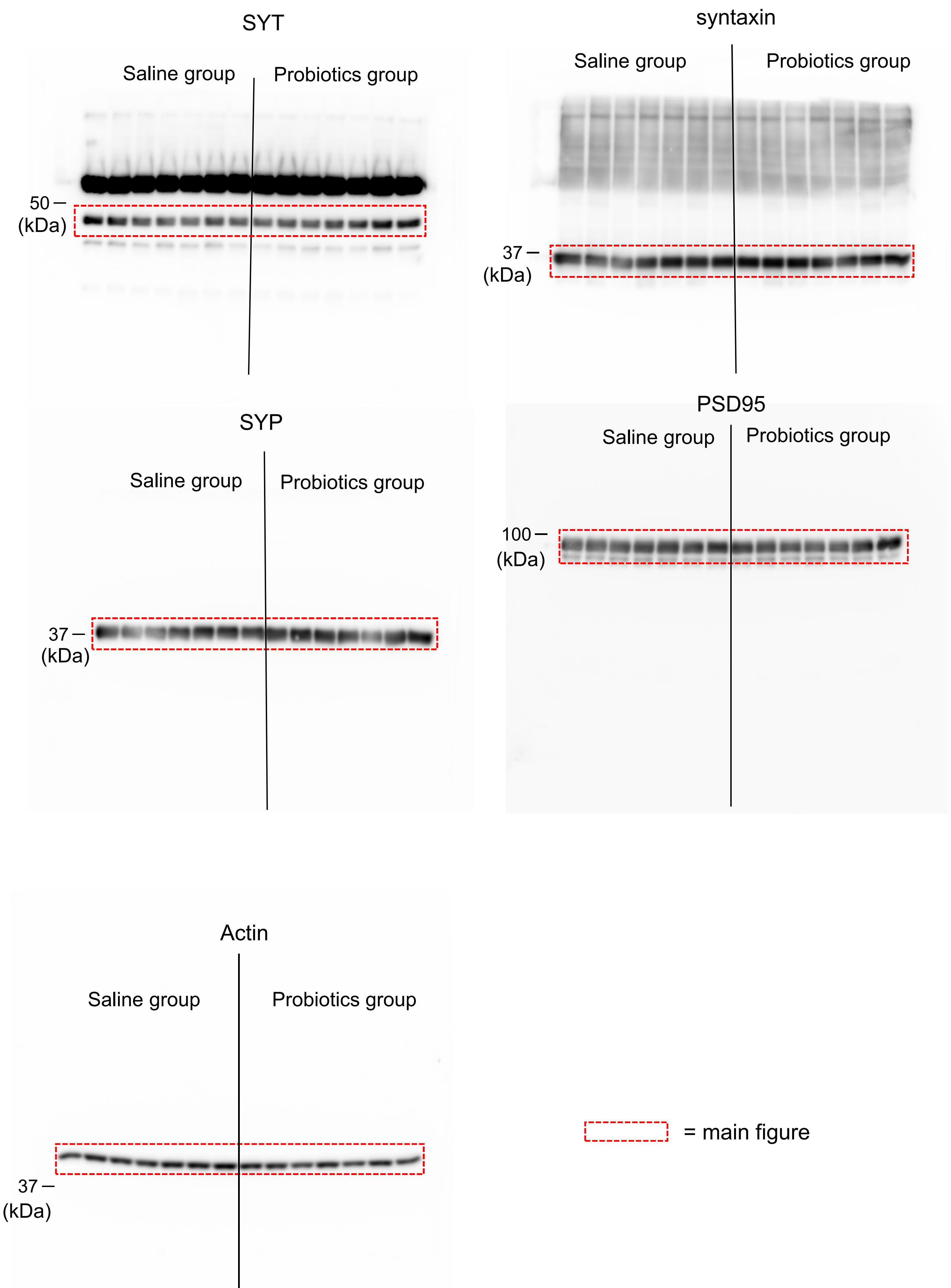
SYT
Saline group
Probiotics group
50 —
(kDa)
syntaxin
Saline group
Probiotics group
37 —
(kDa)
SYP
Saline group
Probiotics group
37 —
(kDa)
PSD95
Saline group
Probiotics group
100 —
(kDa)
Actin
Saline group
Probiotics group
37 —
(kDa)
= main figure

Figure 6 B

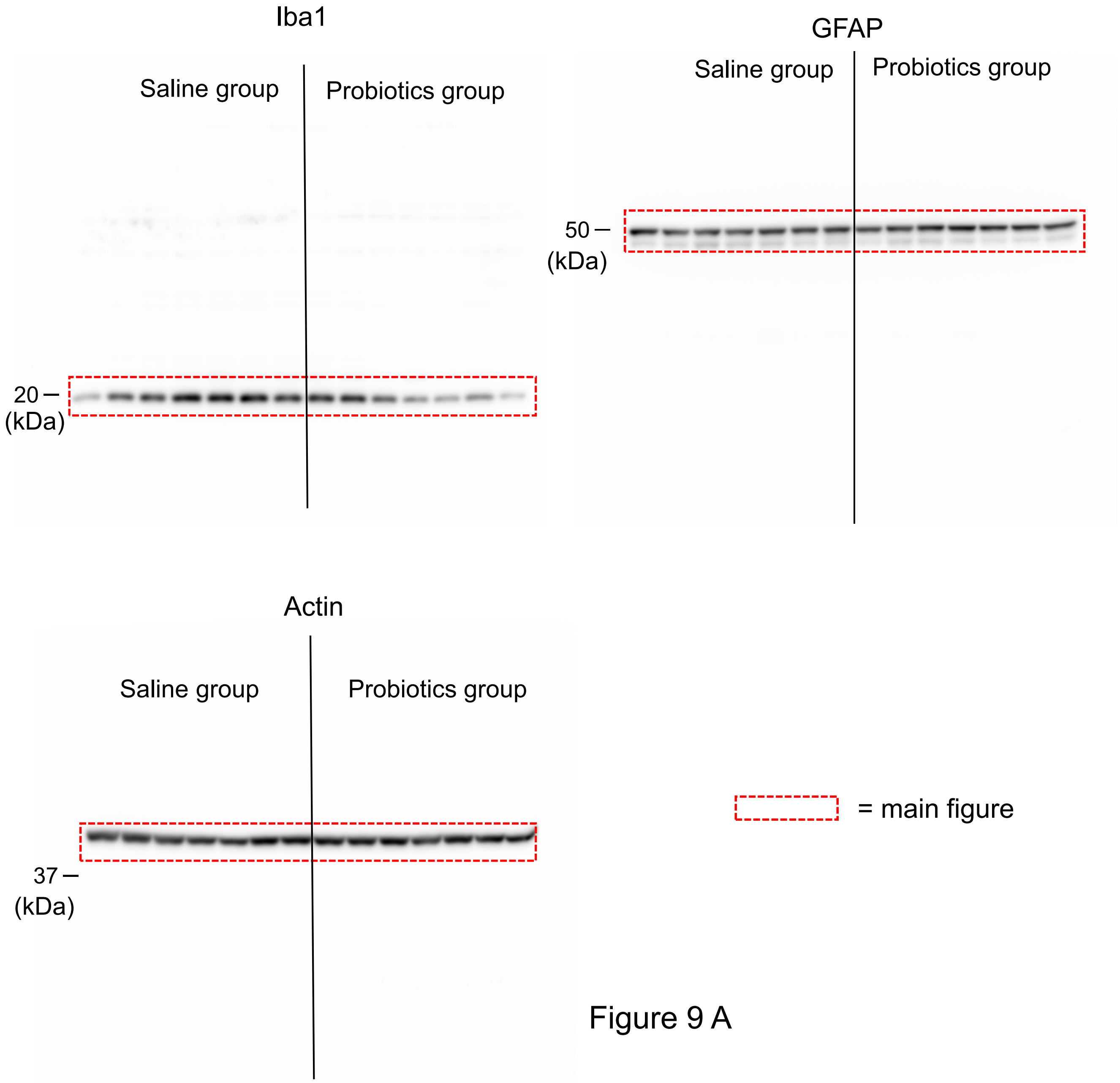
Iba1
Saline group
Probiotics group
20
(kDa)
GFAP
Saline group
Probiotics group
50
(kDa)
Actin
Saline group
Probiotics group
37
(kDa)
= main figure

Figure 9 A

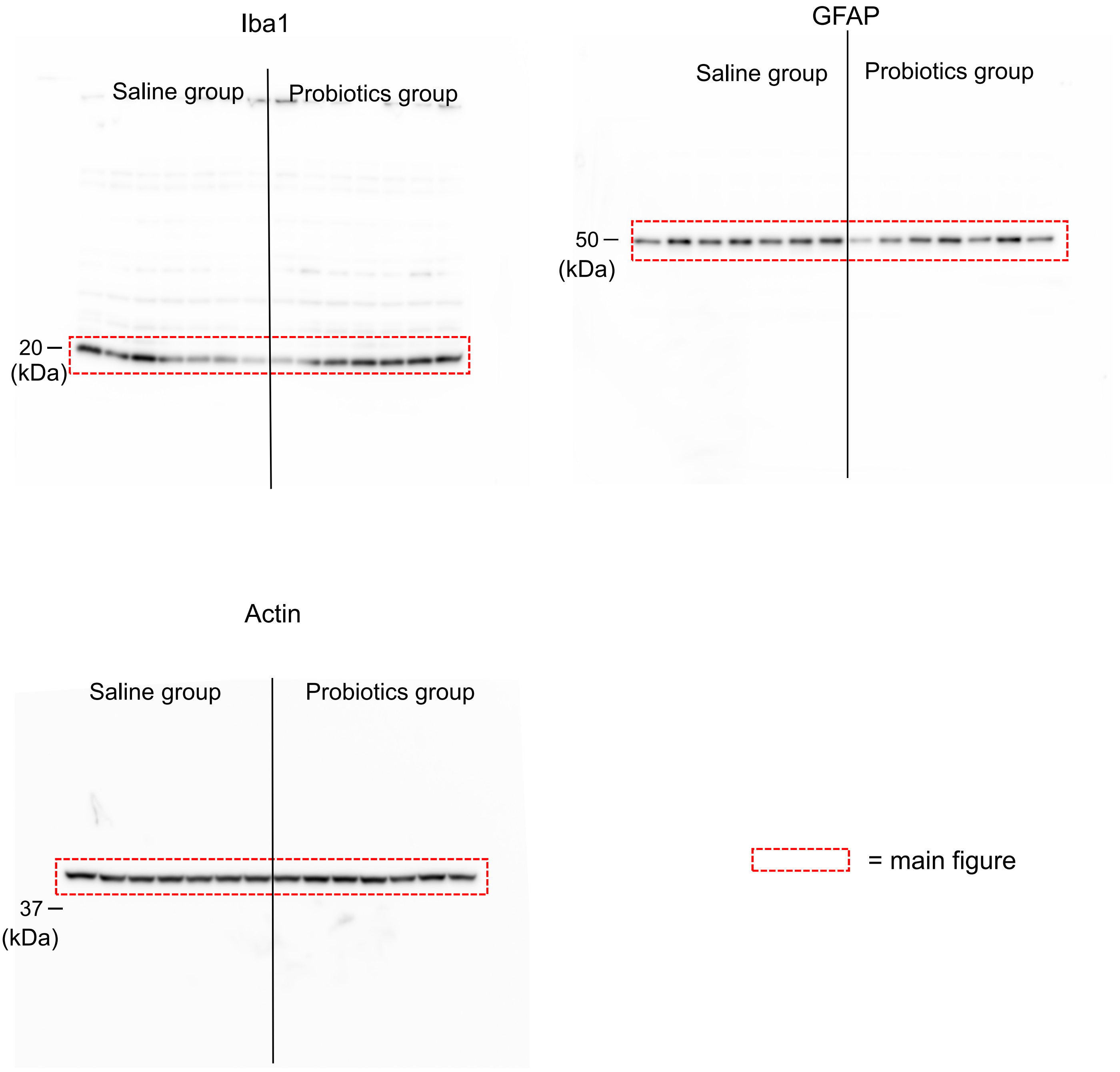
Iba1
Saline group
Probiotics group
20
(kDa)
GFAP
Saline group
Probiotics group
50
(kDa)
Actin
Saline group
Probiotics group
37
(kDa)
= main figure

Figure 9 B

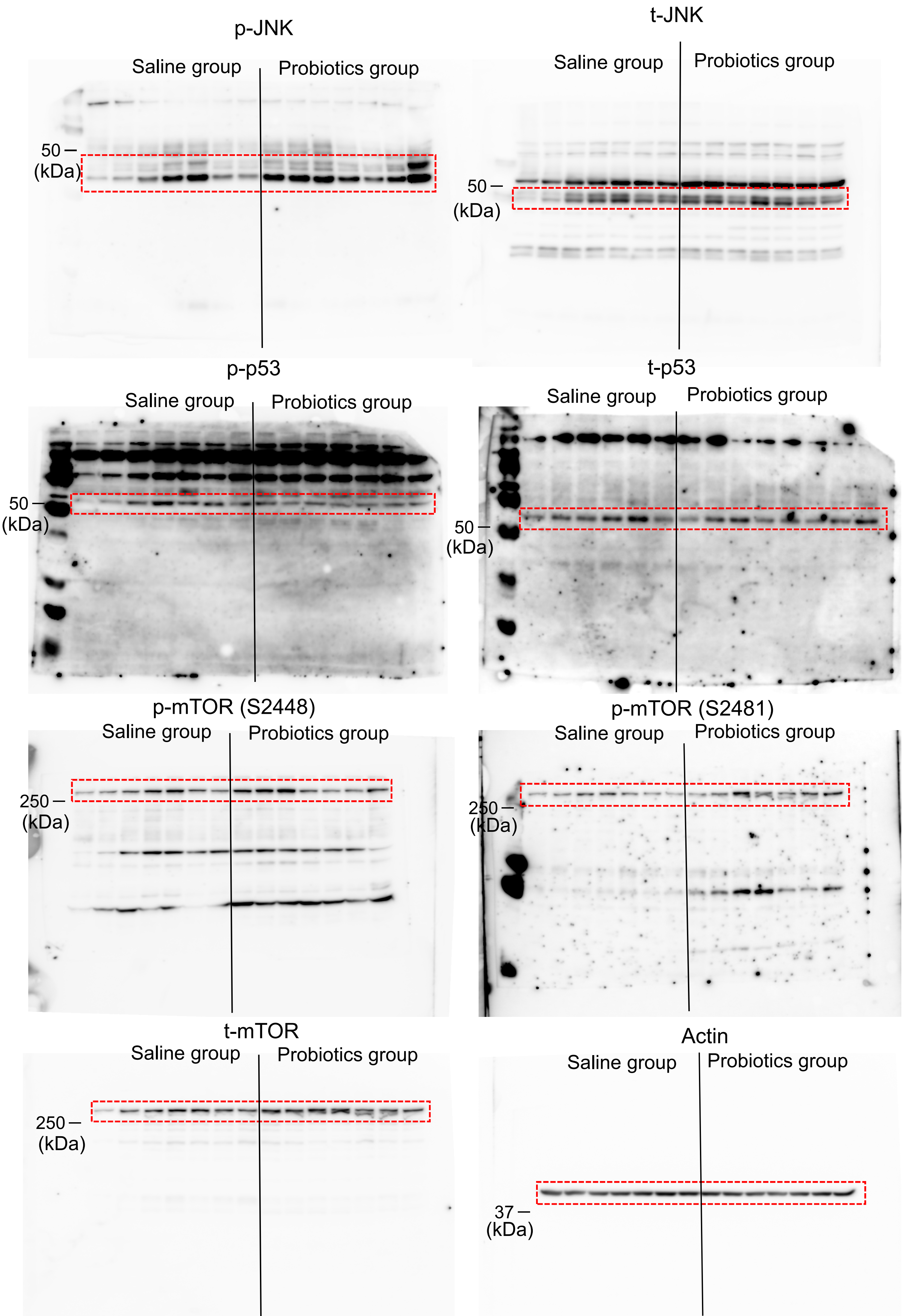

Supplementary Figure S1A

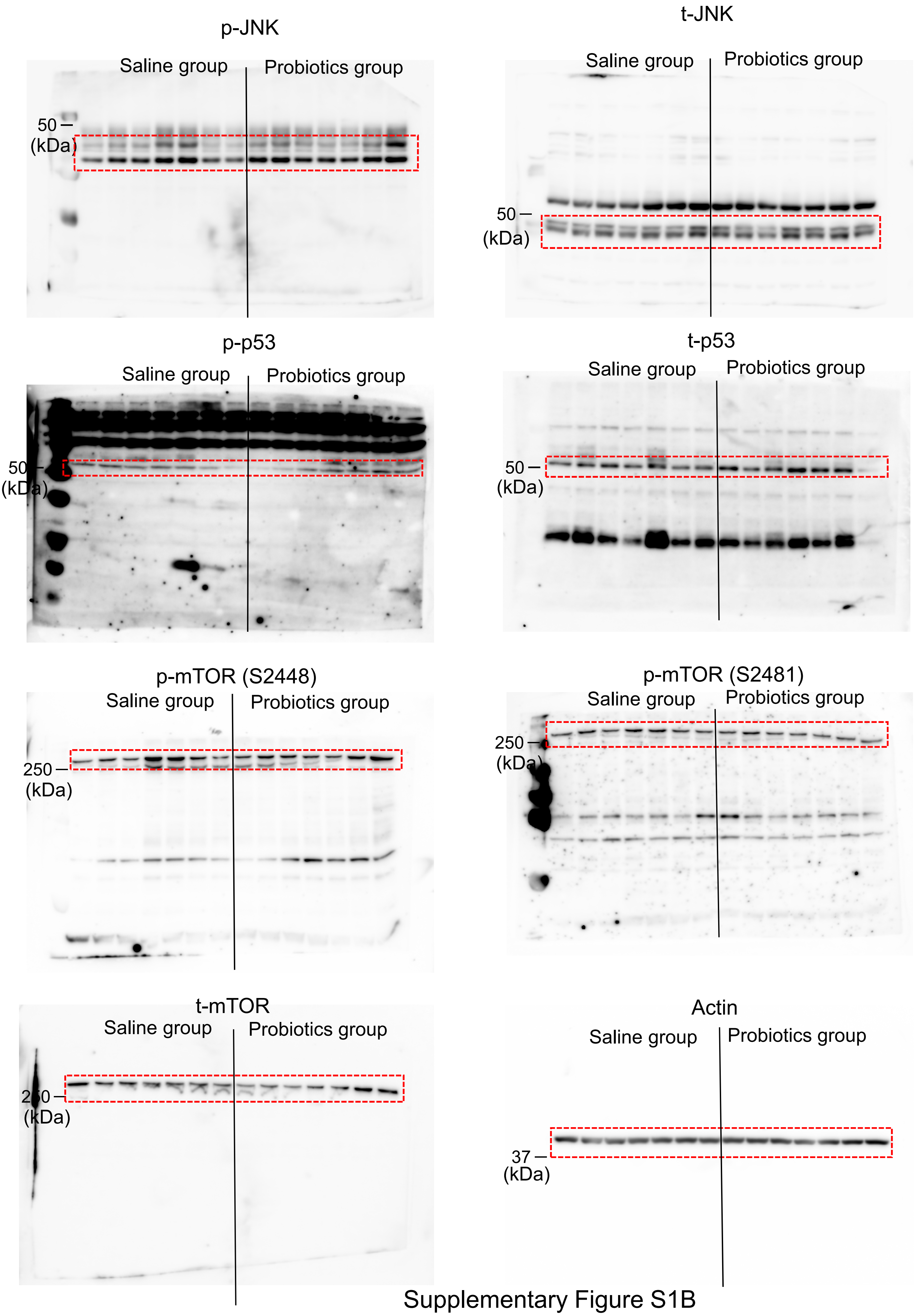

Supplementary Figure S1B
